# Changes in cognitive processes and coping strategies precede changes in symptoms during cognitive therapy for posttraumatic stress disorder

**DOI:** 10.1016/j.brat.2023.104407

**Published:** 2023-10

**Authors:** Milan Wiedemann, Magdalena Janecka, Jennifer Wild, Emma Warnock-Parkes, Richard Stott, Nick Grey, David M. Clark, Anke Ehlers

**Affiliations:** a University of Oxford, Oxford, UK; b Oxford Health NHS Foundation Trust, Oxford, UK; c Icahn School of Medicine at Mount Sinai, Department of Psychiatry, New York, USA; d King's College London, London, UK; e South London and Maudsley NHS Foundation Trust, London, UK; f Sussex Partnership NHS Foundation Trust, Worthing, UK

**Keywords:** PTSD, Cognitive therapy, Change processes, Appraisals, Memory, Coping, Structural equation model

## Abstract

Theories of posttraumatic stress disorder (PTSD) highlight the role of cognitive and behavioral factors in its development, maintenance, and treatment. This study investigated the relationship between changes in factors specified in Ehlers and Clark's (2000) model of PTSD and PTSD symptom change in 217 patients with PTSD who were treated with cognitive therapy for PTSD (CT-PTSD) in routine clinical care. Bivariate latent change score models (LCSM) of session-by-session changes in self-report measures showed that changes in PTSD symptoms were preceded by changes in negative appraisals, flashback characteristics of unwanted memories, safety behaviours, and unhelpful responses to intrusions, but not vice versa. For changes in trauma memory disorganization and PTSD symptoms we found a bidirectional association. This study provides evidence that cognitive and behavioral processes proposed in theoretical models of PTSD play a key role in driving symptom improvement during CT-PTSD.

## Highlights

• Our study found that cognitive and behavioural processes play a key role in symptom improvements during cognitive therapy for posttraumatic stress disorder.
• This supports psychological interventions that target the modification of negative appraisals, the elaboration of trauma memories, and work on giving up unhelpful coping strategies.

Trauma-focused cognitive-behavioral therapies for posttraumatic stress disorder (PTSD) are effective (for reviews see Cusack et al., 2016; International Society for Traumatic Stress Studies, 2020; Kline, Cooper, Rytwinksi, & Feeny, 2018; Mavranezouli et al., 2020; National Institute for Health and Care Excellence, 2018) and are recommended as first-line interventions in international treatment guidelines (American Psychological Association, 2017; International Society for Traumatic Stress Studies, 2020; National Institute for Health and Care Excellence, 2018). These treatments show significant overlap in treatment goals and procedures (Schnyder et al., 2015). However, there is as yet sparse empirical evidence on the cognitive and behavioral processes that drive symptom change during treatment.

Several theories of PTSD emphasize the role of cognitive and behavioral processes in the development and maintenance of PTSD. For example, according to Ehlers and Clark's (2000) cognitive model people with PTSD perceive a sense of internal or external current threat due to (1) negative appraisals (personal meanings) of the traumatic event or its aftermath and (2) the disjointed nature of trauma memories, which in combination with perceptual priming and associative learning, leads to easy triggering of reexperiencing symptoms. Individuals with PTSD respond to the perceived threat and reexperiencing symptoms with a range of unhelpful cognitive and behavioral coping strategies that maintain the problem, in particular suppression of memories and thoughts about the trauma, rumination, emotional numbing, and excessive precautions (safety behaviors). These maintain PTSD symptoms either directly or by preventing change in appraisals and trauma memories. Other models of PTSD have also highlighted the role of appraisals (e.g., Foa & Riggs, 1993; Resick & Schnicke, 1992), memory processes (e.g., Brewin, Gregory, Lipton, & Burgess, 2010; Foa & Riggs, 1993), and unhelpful coping strategies (e.g., Foa & Riggs, 1993; Resick & Schnicke, 1992).

There is evidence for the role of these factors from prospective studies of trauma survivors and some initial studies investigating changes with treatment. Prospective studies have found that negative appraisals about the self or the world following trauma (e.g., ‘*I am inadequate*’, ‘*I have to be on guard all the time*’) predict PTSD (e.g., Dunmore, Clark, & Ehlers, 2001; Beierl, Böllinghaus, Clark, Glucksman, & Ehlers, 2019). Most trauma-focused psychological therapies for PTSD aim to change negative appraisals (Schnyder et al., 2015). A meta-analysis of 16 randomized controlled trials with a total of 994 participants highlighted that psychological therapies for PTSD are efficacious in reducing negative trauma-related appraisals (Diehle, Schmitt, Daams, Boer, & Lindauer, 2014). As McNally and Woud (2019) point out, these findings are consistent with a link from cognition to symptoms, but the temporal precedence of appraisals needs to be established to consider them a process that drives symptom change.

Brown, Belli, Asnaani, and Foa's (2018) systematic review identified 15 studies of the directionality of changes between PTSD symptoms and negative appraisals during treatment, and 11 of these found that changes in appraisals preceded PTSD symptoms change. Seven studies included multiple assessments of negative appraisals and PTSD symptoms during treatment, five of which showed that session-by-session changes in negative appraisals preceded changes in PTSD symptoms treatment (Cooper, Zoellner, Roy-Byrne, Mavissakalian, & Feeny, 2017b; Kleim et al., 2013; Kumpula et al., 2017; McLean et al., 2019; Zalta et al., 2014). A recent study by Kooistra et al. (2023) found further evidence that improvements in negative appraisals precede subsequent improvements in PTSD symptoms in patients with childhood abuse-related PTSD during prolonged exposure therapy.

Two main aspects of trauma memories have been highlighted in theories of PTSD, the disorganization of intentionally retrieved trauma memories, as evidenced for example in disorganized trauma narratives, and characteristics of involuntary trauma memories such as the extent to which they appear to happen in the ‘here and now’ (Brewin, 2016; Ehlers & Clark, 2000; Foa and Riggs, 1993). There has been a debate about the definition and assessment of aspects of memory disorganization relevant to PTSD (see Brewin, 2016; Ehlers, 2015; Ehlers, Ehring, & Kleim, 2012); nevertheless, the majority of prospective studies found that disorganized or disjointed memories predicted PTSD after trauma (e.g., Beierl et al., 2019; Halligan, Michael, Clark, & Ehlers, 2003). Studies with small sample sizes (*n* = 14 to *n* = 77) provided mixed results on changes in memory disorganization during psychological treatment in adults (Bedard-Gilligan, Zoellner, & Feeny, 2017; Foa, Molnar, & Cashman, 1995; Kindt, Buck, Arntz, & Soeter, 2007; Mundorf & Paivio, 2011; van Minnen, Wessel, Dijkstra, & Roelofs, 2002) as well as children and adolescents (Kangaslampi & Peltonen, 2019; Meiser-Stedman et al., 2017). Mundorf and Paivio (2011) found that narrative incoherence did not consistently improve during treatment but higher scores at pretreatment were associated with less improvement in PTSD symptoms during treatment. Bedard-Gilligan et al. (2017) did not find such an association and also found no association between improvements in memory fragmentation and recovery from PTSD. Different definitions and methods of assessing the extent of trauma memory disorganization (e.g., independent coding of trauma narratives versus self-reported measures) complicate the comparability between studies. Only one study of a small sample of children and adolescents investigated whether changes in memory characteristics during treatment are associated with subsequent changes in PTSD symptoms, but found no evidence supporting this hypothesis (Meiser-Stedman et al., 2017).

Regarding intrusive trauma memories, Michael, Ehlers, Halligan, and Clark (2005) found that while intrusive memories in trauma survivors with and without PTSD have similar features (such as sensory impressions), some characteristics distinguished these groups, and also predicted future PTSD symptoms (e.g., nowness, distress, lack of context, and easy triggering). Hackmann, Ehlers, Speckens, and Clark (2004) and Speckens, Ehlers, Hackmann, and Clark (2006) found that these intrusion characteristics decreased during a course of treatment. However, the temporal relationship between changes in memory characteristics and PTSD symptoms was not assessed.

Prospective studies supported the role of unhelpful coping strategies in predicting the development of PTSD after experiencing a traumatic event (e.g., Beierl et al., 2019; Dunmore et al., 2001; Ehring, Ehlers, & Glucksman, 2008; Michael, Halligan, Clark, & Ehlers, 2007; Murray, Ehlers, & Mayou, 2002). Trauma-focused psychological treatments encourage patients to drop unhelpful coping strategies, and reductions in use of these strategies have been linked to better treatment outcomes. A treatment study of 95 veterans with PTSD receiving exposure therapy showed that reductions in safety behaviors were associated with lower depression and PTSD symptoms at post-treatment (Goodson & Haeffel, 2018). Brady, Warnock-Parkes, Barker, and Ehlers (2015) analyzed video tapes of an early session of cognitive therapy for PTSD in 58 patients and found that higher levels of rumination and worrying during that session were associated with worse treatment outcomes. A better understanding of how changes in common unhelpful coping strategies are related to changes in PTSD symptoms is needed to evaluate their role in clinical improvement.

Thus, there is initial evidence that change in negative appraisals of the traumas drives PTSD symptom change in trauma-focused cognitive-behavioral treatments, but the evidence for the role of changes in memory characteristics or cognitive and behavioral coping strategies remains limited. Further research is needed to investigate cognitive and behavioral factors that are involved in clinical improvement (Brown, Belli, Asnaani, & Foa, 2018; Cooper et al., 2017a; McNally & Woud, 2019). Furthermore, although most studies used advanced statistical techniques (e.g., lagged mixed-effects models or bivariate latent growth modeling) to investigate longitudinal associations between negative appraisals and PTSD symptoms during treatment, direct tests of whether session-by-session *changes* in theory-derived candidate processes precede *changes* in PTSD symptoms are as yet lacking.

The present study investigated changes in trauma-related negative appraisals, trauma memory characteristics, and cognitive and behavioral coping strategies, and their temporal relationship to changes in PTSD symptoms over the course of cognitive therapy for PTSD (CT-PTSD), one of the evidence-based trauma-focused cognitive behavioral therapy programmes recommended as a first-line intervention for PTSD (American Psychological Association, 2017; National Institute for Health and Care Excellence, 2018; International Society of Traumatic Stress Studies, 2020). This treatment builds on Ehlers and Clark's (2000) cognitive model of PTSD and has been shown to be efficacious in randomized controlled trials (e.g., Ehlers, Clark, Hackmann, McManus, & Fennell, 2005; Ehlers et al., 2003, 2014) and effective in routine clinical care (e.g., Ehlers et al., 2013). CT-PTSD aims to reduce the sense of current threat by changing negative appraisals, updating trauma memories, and dropping unhelpful coping strategies (Ehlers et al., 2005; Ehlers and Wild, 2015). We therefore hypothesized that changes in these processes precede changes in PTSD symptoms, building on Kleim et al. (2013) who found that improvements in appraisals predicted subsequent symptom reduction in CT-PTSD, but not vice versa.

## Methods

### Participants

This study is a secondary analysis of data drawn from a cohort study of 343 consecutive patients (Ehlers et al., 2023). Patients met criteria for PTSD as assessed by the Structured Clinical Interview for DSM-IV (SCID; First, Gibbon, Spitzer, Williams, & Benjamin, 1997) who were treated with CT-PTSD in routine clinical care. Outcomes were monitored for all patients who started treatment for PTSD in a National Health Service outpatient clinic serving a diverse catchment area in South London between June 2009 and March 2013. Ethical approval was granted by the local research ethics committee.

To ensure that multiple change scores could be calculated and patients had received at least some of core therapy procedures, we included patients who provided data for PTSD symptoms and at least one of the process measures derived from Ehlers and Clark's (2000) model (see the ‘Measures’ section below) for at least 5 of the first 10 sessions (*n* = 217 patients), see Table 1 for patient characteristics. Participants with sufficient data had attended more treatment sessions than those with insufficient data (*M* = 11.04; *SD* = 4.32 vs *M* = 6.98; *SD* = 5.34). They did not differ in sex, relationship status, education, or type of main traumatic event, but were more likely to be from a White ethnic background (65.0% vs 48.4%), employed (47.5% vs 31%), and have a higher level of education (University degree: 31.3% vs 14.3%).

**Table 1:** *Demographic and clinical characteristics* (*n* = 217).

Variable	*n*	%	*M (SD)*
Age (in years)	217		37.47 (10.91)
Months since traumatic event	216		53.38 (80.64)
**Sex**			
Female	120	55.3%	
Male	97	44.7%	
**Ethnicity**			
Black	51	23.5%	
White	141	65.0%	
Indo-Asian	11	5.1%	
Other	14	6.5%	
**Relationship**			
Married/Cohabiting	86	39.6%	
Divorced/Separated/Widowed	23	10.6%	
Never married	100	46.1%	
No information	8	3.7%	
**Education**			
University	68	31.3%	
A-levels (national exam age 18)	30	13.8%	
GCSE (national exam age 16)	48	22.1%	
Other	29	13.4%	
No information	42	19.4%	
**Employment**			
Employed/Self-employed	103	47.5%	
Sick leave	12	5.5%	
Disability/Retired	10	4.6%	
Unemployed	69	31.8%	
Student	9	4.1%	
No information	14	6.5%	
**Type of main traumatic event**			
Interpersonal violence	135	62.2%	
Accident or disaster	44	20.3%	
Death or harm to others	27	12.4%	
Other	11	5.1%	

*Note. n* = Number of patients. % = Percentage of total sample in this study.

For comparability with Kleim et al. (2013) and to reduce the overall rate of missing data, only responses from the questionnaires filled in during the initial 10 weeks of the therapy were used for the current analysis.

### Treatment

Patients received a course of CT-PTSD (Ehlers et al., 2005). CT-PTSD aims to reduce the patient's sense of current threat by (1) changing excessively negative appraisals (personal meanings) of the trauma and its consequences, (2) elaborating and updating the memories for the worst moments of the trauma(s) with information that gives them a less threatening meaning, (3) discriminating triggers of intrusive memories, and (4) changing behaviors and cognitive processes that maintain PTSD. The therapy is tailored to each patient based on the individual case formulation, with the relative weight given to each treatment procedure differing between the individuals. Treatment started with the individual formulation, reclaiming your life assignments and usually the memory updating procedure.

### Therapists

The therapists were qualified clinical psychologists, psychiatrists, nurse therapists, or trainees in these professions or cognitive behavior therapy. All therapists had completed at least basic training in cognitive behavior therapy and a workshop on CT-PTSD. Staff and trainee therapists delivered the treatment, with the majority of patients being treated by staff therapists. All cases were discussed in weekly supervision meetings and trainees also received individual case supervision to ensure fidelity of treatment delivery.

### Measures

Patients were asked to complete PTSD symptom and process measures before each weekly treatment session. The questionnaires covered the time frame of the previous week. For the current analyses we used mean item scores for each questionnaire to assist in the interpretation of therapeutic improvements and reduce the variance of the scores to facilitate the estimation of parameters. The item wordings for the therapy process measures used in this study are available at https://oxcadatresources.com/questionnaires-ptsd/.

#### PTSD symptoms

The Posttraumatic Diagnostic Scale (PDS; Foa, Cashman, Jaycox, & Perry, 1997) assessed the PTSD symptoms specified in DSM-IV (American Psychiatric Association, 2000). Patients were asked to rate how much they were bothered by each of the 17 symptoms in the past week on a scale from 0 (*Not at all*) to 3 (*5 or more times a week*). The internal consistency at baseline was Cronbach's α = 0.89.

#### Process measures

These covered central factors in the maintenance of PTSD specified in Ehlers and Clark's model (2000), appraisals (1 scale), trauma memory characteristics (disorganization of memory recall and flashback quality of unwanted memories) and unhelpful coping (unhelpful responses to trauma memories and safety behaviours). The scales have shown good psychometric properties in previous studies, including expert ratings on content validity, correlations with questionnaires measuring related constructs and predictive validity in that they have shown to predict PTSD after trauma in a range of longitudinal studies (e.g., Beierl et al., 2019; Ehring et al., 2008; Wild et al., 2016) and shown to mediate differences in outcome between trauma-focused and nontrauma focused internet-delivered treatment of PTSD (Ehlers et al., 2023).

#### Negative appraisals

Negative trauma-related appraisals were assessed with a short 20-item version of the Posttraumatic Cognitions Inventory (PTCI-s; Ehlers, 2023). Patients rated how much they agreed with the statements representing a range of cognitive themes: vulnerable self, self-criticism, overgeneralized danger, preoccupation with unfairness, perceived permanent change, alienation, hopelessness and negative view of body, each from 1 (*Totally disagree*) to 7 (*Totally agree*). The internal consistency at baseline was Cronbach's α = 0.91.

#### Memory disorganization

Disorganization of patients' trauma memories was assessed using a 5-item version of the Trauma Memory Questionnaire (e.g., “My memory of the trauma was muddled”, TMQ; adapted from Halligan et al., 2003). Patients rated the extent of the disorganization of their memories of the traumatic experiences on 5 items ranging from 0 (*Not at all*) to 4 (*Very strongly*). The internal consistency at baseline was Cronbach's α = 0.84.

#### Flashback characteristics

Patients reported characteristics of their intrusive trauma memories on the Unwanted Memories Questionnaire (UMQ; adapted from Hackmann et al., 2004). Patients were asked to report the perceived nowness, disjointedness, sense of reliving, distress and the ease in which their main intrusions were triggered. Each item ranged from 0 (*Not at all*) to 100 (*Very strongly*). The scores of this measure were divided by 10 to reduce the variance and facilitate parameter estimation in data analyses. The internal consistency at baseline was Cronbach's α = 0.82.

#### Unhelpful responses to intrusions

These were assessed with a short 12-item version of the Responses to Intrusions Questionnaire (RIQ-s; adapted from Clohessy and Ehlers, 1999; Murray et al., 2002). Patients were asked to rate to what extent they responded to unwanted memories with effortful suppression (e.g., “I try to push them out of my mind”), rumination (e.g., “I dwell on how I used to be before the event”), and emotional numbing (e.g., “I numb my feelings”) on a scale from 0 (*Never*) to 3 (*Always*). The internal consistency at baseline was Cronbach's α = 0.81.

#### Safety behaviors

Common general safety behaviors were assessed using a short 7-item version of the Safety Behaviours Questionnaire (SBQ-s; adapted from Dunmore, Clark, & Ehlers, 1999; Dunmore et al., 2001). Patients were asked to indicate how often they take extra precautions (e.g., “I overprotect those close to me”) on a scale from 0 (*Never*) to 3 (*Always*). The internal consistency at baseline was Cronbach's α = 0.85.

### Statistical analysis

All analyses were performed in R (Version 4.0.2; R Core Team, 2018) through R Studio IDE (RStudio Team, 2020). Univariate and bivariate latent change score models (LCSM) were estimated using the R package *lavaan* (Version 0.6.7; Rosseel, 2012) and model syntax was generated using the R package *lcsm* (Version 0.1.4; Wiedemann, Thew, Kosir, & Ehlers, 2022). All analytical decisions were made a priori following the underlying cognitive model and clinical procedures used in treatment, but were not preregistered. Supporting data cannot be made available as patients did not consent to their data being shared. The analysis code is available at https://osf.io/h3v7t.

**Fig. 1:**
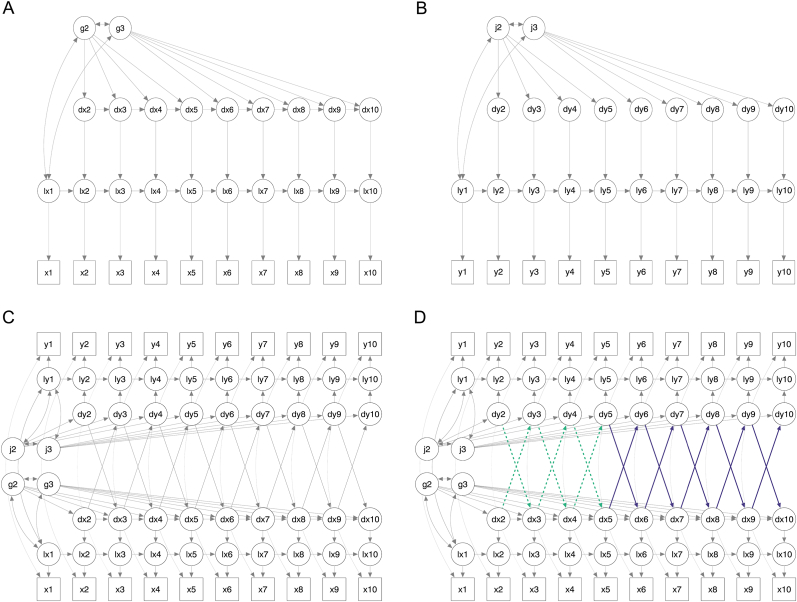
Simplified path diagrams for univariate and bivariate LCSMs. Univariate LCSMs (A) including and (B) not including autoregressions of change scores. Bivariate LCSMs (C) restricting coupling parameters over the entire treatment and (D) restricting coupling parameters for the first (dotted green line) and second (solid purple line) part of treatment. Squares = Observed variables; Circles = Latent variables; Single-headed arrows = Regressions; Double-headed arrows = Covariance. ‘x’ (PTSD symptoms) and ‘y’ (Process measures) represent the measured variables, the prefix ‘l’ indicates the latent construct, and the prefix ‘d’ indicates latent change scores. ‘g’ and ‘j’ represent constant change factors.

First, univariate LCSMs with increasing complexity were fit for PTSD symptoms and each therapy process measure separately to determine how each construct changed during treatment. We assumed longitudinal measurement invariance, i.e., that measures represented the same construct at each assessment. Considering previous findings about early changes in symptoms and cognitive processes during cognitive therapies (e.g., Kleim et al., 2013; Macdonald, Monson, Doron-Lamarca, Resick, & Palfai, 2011) and differences in the therapy techniques used predominantly in early versus later sessions of CT-PTSD (see Ehlers et al., 2005), we tested whether allowing changes in PTSD symptoms and all process measures to be different between the first (changes up to session 5) and second (changes from session 5 onwards) part of therapy improved model fit compared to a constant change throughout therapy. Simplified path diagrams illustrating the best fitting univariate LCSMs for PTSD symptoms and other PTSD therapy process measures are presented in Fig. 1A and B respectively.

Second, we estimated bivariate LCSMs with increasing complexity to evaluate the temporal associations between changes in PTSD symptoms and each cognitive process separately (Grimm, An, McArdle, Zonderman, & Resnick, 2012; McArdle, 2009). The best fitting univariate LCSM for each construct was selected (see Table A2 in Supplemental Online Material) and we tested whether adding lagged coupling parameters between the constructs improved the model fit. To test the hypothesized effect that changes in PTSD symptoms ($\Delta {PTSD\;symptoms}_{\left(t\right)}$) are determined by prior changes in each cognitive process ($\Delta {Cognitive\;process}_{\left(t-1\right)}$) we added the parameter $\xi_{{lag}_{xy}}$ ($\Delta {Cognitive\;process}_{\left(t-1\right)}\to \Delta {PTSD\;symptoms}_{\left(t\right)}$). To contrast this with the alternative explanation – that changes in PTSD symptoms lead to changes in each cognitive process – we also tested the reverse relationship described as parameter $\xi_{{lag}_{yx}}$ ($\Delta {PTSD\;symptoms}_{\left(t-1\right)}\to \Delta {Cognitive\;process}_{\left(t\right)}$) and a bidirectional relationship by adding both parameters $\xi_{{lag}_{xy}}$ and $\xi_{{lag}_{yx}}$. To simplify the model interpretation and permit its full identification, several restrictions were imposed on the univariate and bivariate LCSMs following methodological recommendations (Grimm, Ram, & Estabrook, 2017) and similar clinical studies (Hawley et al., 2017). These included fixing autocorrelations within constructs and covariances of residuals between constructs across time. Lagged coupling parameters were set to equal throughout therapy suggesting that improvement in process measures would predict improvement in symptoms similarly, except for when the therapy content suggested that this effect may act differently during specific parts of the treatment. Addressing negative appraisals, unhelpful responses to intrusive memories (suppression, rumination, numbing) and safety behaviors is a key aim addressed throughout all treatment sessions in CT-PTSD, therefore cross-lagged coupling effects were set to equal over time for these process measures.

Interventions that address trauma memories in CT-PTSD differ between earlier and later sessions. In the early sessions, patients are asked to access the memories of their main trauma in imaginal reliving (visualizing and giving an oral account of what happened moment by moment) or by writing a moment-by-moment trauma narrative, and therapists then guided them to update the meanings of the worst moments. In the later sessions, therapeutic techniques focus on memory triggers and a site visit. These different interventions may have different effects on changes in trauma memories (Ehlers et al., 2005). We therefore allowed the lagged coupling effects between memory characteristics (disorganized memories and flashback characteristics) and PTSD symptoms to vary between the early session changes (changes up to session 5) and subsequent changes (changes from session 5 onwards) – in the following this is referred to as ‘piecewise’. A detailed description of the treatment procedures is described elsewhere (see Ehlers et al., 2005; Ehlers and Wild, 2015). Simplified path diagrams illustrating differences in modelling strategies between memory characteristics and other PTSD therapy process measures are presented in Fig. 1C and D. All models were estimated using the Full Information Maximum Likelihood (FIML) estimator. We conducted likelihood ratio tests for competing models that were nested and also considered different types of absolute and comparative fit indices to determine the best fitting univariate and bivariate LCSMs: Models with smaller values on the Akaike Information Criterion (AIC; Akaike, 1974) and Bayesian Information Criterion (BIC) indicate better model fit, values ≥ 0.95 on the Comparative Fit Index (CFI; Bentler, 1990) and Tucker-Lewis Index (TLI; Tucker & Lewis, 1973) suggest good model fit, and we consider values ≤ 0.10 on the root mean square error of approximation (RMSEA; Steiger & Lind, 1980) to suggest adequate fit (MacCallum, Browne, & Sugawara, 1996).

## Results

### Changes in PTSD symptoms and process measures during therapy

**Fig. 2:**
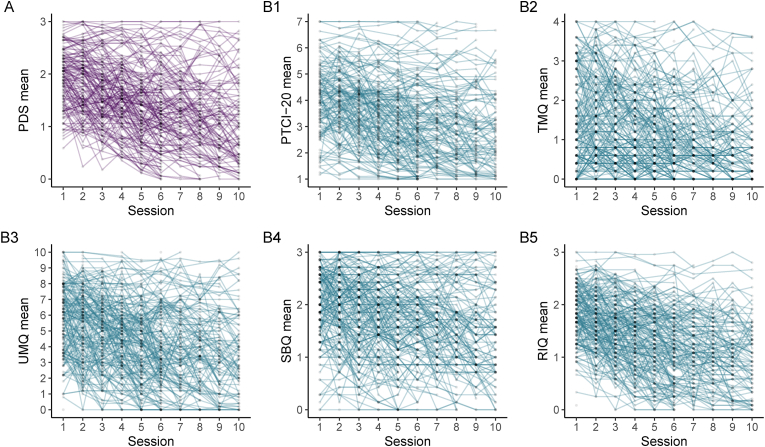
Observed individual trajectories of mean scores of PTSD symptoms and process measures during therapy. For clarity of presentation, data from a random sample of 70 % is shown. (A) PDS = PTSD symptoms; (B1) PTCI = Negative appraisals; (B2) TMQ = Disorganized memories; (B3) UMQ = Flashback characteristics; (B4) SBQ = Safety behaviors; (B5) RIQ = Unhelpful responses to intrusions.

Due to small differences in questionnaire completion, the sample size varies slightly between the analyses for each process measure (*n*_PDS–PTCI_ = 212; *n*_PDS–SBQ_ = 211; *n*_PDS–RIQ_ = 215; *n*_PDS–UMQ_ = 204; *n*_PDS–TMQ_ = 212). Mean scores of PTSD symptoms and all process measures decreased over the first ten therapy sessions (see Fig. 2). Means and standard deviations of all measures across all time points are presented in the Supplemental Online Material (Table A1).

Parameter estimates for the best fitting univariate LCSMs for PTSD symptoms and each process measure, respectively, are presented in the Supplemental Online Material (see Tables A3 and A4) and the fit statistics for all tested univariate models can be found in Table A2. For all measures, except for disorganized memories, the estimates suggest greater improvements in the initial five weeks of therapy (Constant change 1 mean: $\alpha_{g2\;\left(PDS\right)}$ = −0.10 [0.02]; $\alpha_{j2\;\left(PTCI\right)}$ = −0.22 [0.02]; $\alpha_{j2\;\left(TMQ\right)}$ = −0.09 [0.02]; $\alpha_{j2\;\left(UMQ\right)}$ = −0.39 [0.04]; $\alpha_{j2\;\left(RIQ\right)}$ = −0.13 [0.01]; $\alpha_{j2\;\left(SBQ\right)}$ = −0.08 [0.01]), with a slower improvement afterwards (Constant change 2 mean: $\alpha_{g3\;\left(PDS\right)}$ = −0.06 [0.02]; $\alpha_{j3\;\left(PTCI\right)}$ = −0.14 [0.01]; $\alpha_{j3\;\left(TMQ\right)}$ = −0.11 [0.01]; $\alpha_{j3\;\left(UMQ\right)}$ = −0.32 [0.03]; $\alpha_{j3\;\left(RIQ\right)}$ = −0.08 [0.01]; $\alpha_{j3\;\left(SBQ\right)}$ = −0.08 [0.01]). Patients varied significantly in their change scores during the first (Constant change 1 variance: $\sigma_{g2}^{2}$ and $\sigma_{j2}^{2}$) and second (Constant change 2 variance: $\sigma_{g3}^{2}$ and $\sigma_{j3}^{2}$) part of therapy on all measures. Univariate latent change score models also suggested that higher initial scores in therapy process measures correlated with slower improvement in these processes during the first or second part of therapy^1^; we did not find this pattern for PTSD symptoms (see parameter estimates of the covariance between the initial status and change during the first ($\sigma_{g2,lx1}$ and $\sigma_{j2,ly1}$) and second part ($\sigma_{g3,lx1}$ and $\sigma_{j3,ly1}$) of therapy in Tables A2 and A3). The best fitting model for PTSD symptoms also suggested that changes in PTSD symptoms were significantly correlated with subsequent changes in PTSD symptoms (Autoregression of change scores: $\varphi_{x\;\left(PDS\right)}$ = 0.38 [0.16]), i.e., patients with large improvements in symptoms at a certain session also showed large improvements during the following sessions.

### Associations between changes in PTSD symptoms and process measures during therapy

Parameter estimates for the best fitting bivariate LCSMs between PTSD symptoms and each process measure, respectively, are presented in Table 2, model fit statistics of all tested bivariate models are shown in the Supplemental Online Material (Table A5). For all models the covariances of residuals between PTSD symptoms and the process measures ($\sigma_{su}$) were significant. The covariances of the intercepts between PTSD symptoms and process measures ($\sigma_{ly1,lx1}$) were also significant in all models, indicating that patients who reported higher levels of PTSD symptoms at the beginning of treatment also showed higher scores on all process measures. The $\xi_{{lag}_{xy}}$ parameter estimates in Table 2 test the hypothesized effect that changes in PTSD symptoms are determined by prior changes in the respective cognitive process.

**Table 2:** Parameter estimates for *best fitting bivariate LCSMs of the lagged associations between changes in process measures and PTSD symptoms*.

Parameter	PTCI - PDS			TMQ - PDS			UMQ - PDS			RIQ - PDS			SBQ - PDS		
	*EST* (*SE*)	*p*	*EST*_*STD*_	*EST* (*SE*)	*p*	*EST*_*STD*_	*EST* (*SE*)	*p*	*EST*_*STD*_	*EST* (*SE*)	*p*	*EST*_*STD*_	*EST* (*SE*)	*p*	*EST*_*STD*_
$Covariance\;of\;residuals\;x\;and\;y\;\left(\sigma_{su}\right)$	0.04 (0.01)	<.001	0.36	0.03 (0.01)	<.001		0.10 (0.01)	<.001	0.38	0.02 (0.00)	<.001	0.36	0.02 (0.00)	<.001	0.25
$Covariance\;of\;initial\;status\;x\;and\;y\;\left(\sigma_{ly1,lx1}\right)$	0.53 (0.06)	<.001	0.78	0.28 (0.04)	<.001		0.80 (0.09)	<.001	0.79	0.23 (0.03)	<.001	0.76	0.22 (0.03)	<.001	0.62
**Changes session 1 to 10 (Δ)**															
$\Delta {Cognitive\;Process}_{t-1}\to \Delta {PTSD}_{t}\;\left(\xi_{{lag}_{xy}}\right)$	0.52 (0.24)	.031	0.83	–	–	–	–	–	–	1.09 (0.35)	.002	0.85	0.85 (0.15)	<.001	0.76
$\Delta {PTSD}_{t-1}\to \Delta {Cognitive\;Process}_{t}\;\left(\xi_{{lag}_{yx}}\right)$	0.60 (0.56)	.277	0.29	–	–	–	–	–	–	0.10 (0.13)	.469	0.08	–	–	–
**Changes session 1 to 10 (Δ**_**1**_**) and 5 to 10 (Δ**_**2**_**)**															
$\Delta_{1}{Cognitive\;Process}_{t-1}\to \Delta_{1}{PTSD}_{t}\;\left(\xi_{1{lag}_{xy}}\right)$	–	–	–	0.13 (0.05)	.013	0.35	0.35 (0.12)	.004	0.97	–	–	–	–	–	–
$\Delta_{2}{Cognitive\;Process}_{t-1}\to \Delta_{2}{PTSD}_{t}\;\left(\xi_{2{lag}_{xy}}\right)$	–	–	–	0.06 (0.09)	.533	0.10	0.48 (0.16)	.003	1.53	–	–	–	–	–	–
$\Delta_{1}{PTSD}_{t-1}\to \Delta_{1}{Cognitive\;Process}_{t}\;\left(\xi_{1{lag}_{yx}}\right)$	–	–	–	0.72 (0.11)	<.001	0.19	1.40 (0.85)	.099	0.29	–	–	–	–	–	–
$\Delta_{2}{PTSD}_{t-1}\to \Delta_{2}{Cognitive\;Process}_{t}\;\left(\xi_{2{lag}_{yx}}\right)$	–	–	–	0.75 (0.16)	<.001	0.66	0.18 (0.98)	.850	0.08	–	–	–	–	–	–

*Note.* EST = Unstandardized estimated parameter; SE = Standard error; EST_STD_ = Standardized estimated parameter (completely standardized solution); PDS = PTSD symptoms; PTCI = Negative appraisals; TMQ = Disorganized memories; UMQ = Flashback characteristics; RIQ = Unhelpful responses to intrusions; SBQ = Safety behaviors. - indicates parameter was not estimated. The $\xi_{{lag}_{xy}}$ parameter estimates test the hypothesized effect that changes in PTSD symptoms ($\Delta {PTSD\;symptoms}_{\left(t\right)}$) are determined by prior changes in each cognitive process ($\Delta {Cognitive\;process}_{\left(t-1\right)}$). For trauma memory characteristics this is tested during the first ($\xi_{{1\;lag}_{xy}}$) and second ($\xi_{2\;{lag}_{xy}}$) part of therapy.

### Negative appraisals - PTSD symptoms

The best fitting model included bidirectional coupling parameters ($\chi^{2}$ = 410, CFI = 0.960, TLI = 0.963, RSMEA = 0.069, AIC = 4,465, BIC = 4549). Improvements in negative appraisals predicted improvements in PTSD symptoms in the following session ($\xi_{lag_{xy}}$ = 0.52, SE = 0.24, *p* = .031). To interpret this unstandardized effect the units of the measures that were used in the analysis need be considered. The parameter $\xi_{lag_{xy}}$ = 0.52 indicates that a one-unit improvement in the PTCI mean (ranged from 1 to 7) was associated with an improvement of 0.52 on the PDS mean (ranged from 0 to 3) in the following session. This represents a change of 17.3% in the total sum score of the PDS (ranged from 0 to 51) which translates to an 8.8 points improvement in PTSD symptoms. Therefore, if patients reduced their conviction in their unhelpful appraisals, for example on average from 6 (*Agree very much*) to 5 (*Agree slightly*) on all items, this one-unit improvement in the PTCI mean would lead to an 8.8 point improvement on the PDS sum score in the following session. In contrast, changes in PTSD symptoms did not significantly predict subsequent changes in negative appraisals ($\xi_{lag_{yx}}$ = 0.60, SE = 0.56, *p* = .277).

### Disorganized recall and flashback characteristics - PTSD symptoms

For memory disorganization, the best fitting model included piecewise bidirectional coupling parameters ($\chi^{2}$ = 364, CFI = 0.964, TLI = 0.966, RSMEA = 0.061, AIC = 4,214, BIC = 4304). Lagged coupling effects indicated that changes in disorganized memories predicted subsequent changes in PTSD symptoms for the early sessions of therapy ($\xi_{1{lag}_{xy}}$ = 0.13, *SE* = 0.05, *p* = .013) that included reliving or writing a trauma narrative and memory updating, but not during later sessions. Lagged coupling effects in the other direction indicated that changes in PTSD symptoms predicted subsequent changes in disorganized memories during the early as well as subsequent sessions of therapy ($\xi_{1{lag}_{yx}}$ = 0.72, *SE* = 0.11, *p* < .001, $\xi_{2{lag}_{yx}}$ = 0.75, *SE* = 0.16, *p* < .001).

For flashback characteristics, the best fitting model also included bidirectional coupling parameters ($\chi^{2}$ = 356, CFI = 0.965, TLI = 0.967, RSMEA = 0.061, AIC = 6,664, BIC = 6754). Lagged coupling effects indicated that changes in flashback characteristics predicted subsequent changes in PTSD symptoms in both early sessions ($\xi_{1{lag}_{xy}}$ = 0.35, *SE* = 0.12, *p* = .004) and later sessions ($\xi_{2{lag}_{xy}}$ = 0.48, *SE* = 0.16, *p* = .003) during therapy. In contrast, there were no significant effects of changes in PTSD symptoms on changes in flashback characteristics for early or later sessions.

### Unhelpful responses to intrusions and safety behaviors - PTSD symptoms

For responses to intrusions, the best fitting model included bidirectional coupling effects ($\chi^{2}$ = 373, CFI = 0.965, TLI = 0.968, RSMEA = 0.062, AIC = 2,414, BIC = 2498). Changes in responses to intrusions predicted subsequent changes in PTSD symptoms in the following session ($\xi_{lag_{xy}}$ = 1.09, *SE* = 0.35, *p* = .002). In contrast, changes in PTSD symptoms did not significantly predict subsequent changes in responses to intrusions ($\xi_{lag_{yx}}$ = 0.10, *SE* = 0.13, *p* = .469).

For safety behaviors, the best fitting model included only the coupling effect of $\Delta {Safety\;behaviors}_{t-1}\to \Delta {PTSD\;symptoms}_{t}$ ($\chi^{2}$ = 389, CFI = 0.959, TLI = 0.963, RSMEA = 0.065, AIC = 3,064, BIC = 3144). Changes in safety behaviors were significantly associated with changes in PTSD symptoms in the following session ($\xi_{lag_{xy}}$ = 0.85, *SE* = 0.15, *p* < .001). Adding the reverse relationship $\xi_{{lag}_{yx}}$ ($\Delta {PTSD\;symptoms}_{\left(t-1\right)}\to \Delta {Cognitive\;process}_{\left(t\right)}$) to the model did not improve the fit, indicating that there is no evidence for an effect of changes in PTSD symptoms predicting subsequent changes in safety behaviors.

## Discussion

This study investigated whether key cognitive and behavioral processes hypothesized by Ehlers and Clark's (2000) model of PTSD drive clinical improvement during CT-PTSD in routine clinical care. Our overall findings were that changes in negative appraisals, memory characteristics, and unhelpful cognitive and behavioral coping strategies preceded subsequent changes in PTSD symptoms. For disorganized memories we only observed this effect early in therapy, and a reverse relationship was found throughout therapy. These findings extend prior research on therapeutic processes in CT-PTSD (Kleim et al., 2013) and demonstrate that the theory-derived cognitive processes that CT-PTSD aims to change play a key role in PTSD symptom improvements during therapy (Ehlers and Clark, 2000; Ehlers et al., 2005).

Our finding that trauma-related negative appraisals precede changes in PTSD symptoms extends Kleim et al.’s (2013) findings, using direct tests of the relationships between session-to-session changes and are consistent with cognitive models of PTSD (e.g., Ehlers & Clark, 2000; Foa & Riggs, 1993; Resick & Schnicke, 1992) and the majority of studies investigating this relationship during the course of other trauma-focused treatments (Brown et al., 2018). This is in line with expert consensus that identifying and modifying trauma-related negative appraisals is a central therapeutic aim in different forms of psychological therapies for PTSD (Schnyder et al., 2015). Importantly, our results showed no evidence for a reverse or bidirectional relationship between PTSD symptoms and appraisals in our sample, replicating Kleim et al.’s (2013) findings and most studies that were reviewed by Brown et al. (2018). However, two studies found evidence for a reciprocal relationship between appraisal change and PTSD improvement during prolonged exposure therapy (Kooistra et al., 2023; McLean, Su, & Foa, 2015). Discrepancies may be due to differences in the time intervals between the assessment points, as longer intervals between the measurements may have obscured finer-grain temporal effects. However, this highlights that differences in the temporal relationships between changes in PTSD symptoms and negative cognitions need to be further evaluated.

In line with our hypothesis and research investigating pre-to post-treatment changes in different aspects of trauma narratives (e.g., Mundorf & Paivio, 2011) we found evidence that changes in trauma memory disorganization through the elaboration of what happened during the trauma in the first sessions of therapy led to subsequent improvements in PTSD symptoms. We also found evidence that changes in PTSD symptoms were driving subsequent changes in memory disorganization throughout therapy. This would suggest that improvements in some aspects of memory disorganization are preceded by improvements in PTSD symptoms. A possible explanation may be that reductions in PTSD symptoms include the reduction in cognitive avoidance, which may allow patients to engage more with their trauma memories. This may facilitate further improvements in memory disorganization in later parts of the treatment and explain the bidirectional relationship observed in our sample. To our knowledge this is the first study to investigate lagged effects between disorganized trauma memories and PTSD symptoms during psychological therapy for adults with PTSD. Our results provide initial evidence for a bidirectional effect and suggest that the effect of changes in disorganization on subsequent symptom change depends on the treatment procedures, and was only found when techniques that facilitate memory elaboration such as imaginal reliving and writing a moment-by-moment narrative were used, and meanings of particularly distressing moments were updated. It is also possible that not all aspects of disorganization are equally relevant for stimulating PTSD symptom change, in line with the inconsistent findings in the literature for some self-report measures of disorganization. Further refinements of measures may generate more consistent results. For example, Sachschal, Woodward, Wichelmann, Haag, and Ehlers (2019) distinguished between problems in recall and memory disjointedness in an analogue study with healthy participants and found that disjointedness, but not recall, was related to subsequent intrusions and PTSD symptoms and mediated the relationship between cognitive processing during exposure to a trauma film and intrusions.

Extending earlier research showing that specific flashback characteristics of intrusive trauma memories improved during therapy (e.g., Hackmann et al., 2004; Speckens et al., 2006), we found that changes in these characteristics led to subsequent changes in PTSD symptoms throughout therapy. Reductions in flashback characteristics were associated with subsequent improvements in PTSD symptoms, with a similar effect during the initial five sessions of therapy and subsequent sessions. In contrast to our memory disorganization results, we did not find evidence for a reverse relationship of PTSD symptom reduction on flashback characteristics.

In CT-PTSD unhelpful coping strategies are usually addressed through discussions of their advantages and disadvantages and behavioral experiments (see Ehlers et al., 2005). In line with previous prospective studies of trauma survivors that provided evidence for the role of suppression, rumination, and intentional numbing in the development of PTSD (e.g., Beierl et al., 2019; Kleim, Ehlers, & Glucksman, 2012) our results provide initial evidence that changes in these unhelpful responses to intrusions drive subsequent changes in PTSD symptoms during CT-PTSD. Similarly, our results suggest that dropping unhelpful safety behaviors drives subsequent changes in PTSD symptoms, extending previous evidence from a PTSD treatment study (Goodson & Haeffel, 2018). Taken together, the results not only highlight the importance of cognitive processes in clinical improvement, but also highlight the key role of behavioral changes as suggested by cognitive and behavioral models of PTSD (e.g., Ehlers & Clark, 2000; Foa & Riggs, 1993).

Consistent with earlier studies demonstrating that some patients experience significant improvements early during therapy, we also found that PTSD symptoms and therapy process measures improved more during the first part of therapy compared to the second part (Kleim et al., 2013; Macdonald et al., 2011). The slower improvements during the second part of therapy may in part be explained by floor effects as a significant subgroup had minimal symptoms after the early sessions and in part by complex cases, for example those with multiple traumas, comorbidities or social problems, requiring more treatment sessions as their treatment needed to focus on more traumas and/or significant issues other than the traumas. However, our results from univariate LCSMs also highlight that patients varied significantly in their changes during both parts of therapy ($\sigma_{g2}^{2}$, $\sigma_{g3}^{2}$) suggesting that patients improved via different trajectories and that there may be particular subgroups who need further investigation (e.g., Schumm, Walter, and Chard (2013).

Strengths of this study include that our sample included all patients with sufficient data from consecutive referrals, was ethnically diverse, and patients had a wide range of traumas. Therapists with different levels of expertise delivered the therapy in routine clinical care, increasing the generalizability of our findings. We were able to test all key processes of clinical improvement hypothesized by Ehlers and Clark's (2000) cognitive model of PTSD. Although the PTSD therapy processes were correlated with each other and with PTSD symptoms (see Table A6 in Supplemental Online Material) we found evidence for lagged effects in bivariate LCSM analyses. The use of weekly assessment during treatment allowed for a detailed examination of change processes during treatment, however other time intervals and forms of data collection should be explored if important changes in therapy processes are thought to occur during shorter time periods.

This study has several strengths and limitations. Among the strengths was that a consecutive cohort from an ethnically and socioeconomically diverse catchment area was studied and all patients were diagnosed by a validated diagnostic clinical interview (SCID). However, some patient variables like sexual orientation and religion were not assessed by the National Health Service at the time of recruitment. A further limitation was that therapy effects and processes were assessed by self-report. However, self-report versions facilitated the regular completion of the measures. Second, the sample size and analytical method used to investigate lagged effects did not allow for a combined analysis of PTSD symptoms and all therapy process measures or accounting for the nesting of patients within therapists. Third, because this study investigated the therapy processes derived from Ehlers and Clark's (2000) cognitive model of PTSD, the basis of the case formulation in CT-PTSD which guides treatment, some of the investigated processes may be specific to CT-PTSD, although the results may generalize to other trauma-focused treatments with similar treatment goals and procedures (Schnyder et al., 2015). This study was part of a clinical audit and was therefore not resourced for a formal assessment of treatment fidelity by independent raters. However, weekly supervision included watching parts of treatment sessions and detailed case discussions and it is therefore likely that major deviations would have been spotted. Furthermore, low adherence would likely have increased error variance and would not explain the pattern of findings. The results may also be specific to PTSD as the primary outcome measure in this study. Non-specific or common factors (e.g., therapeutic alliance) and alternative outcome measures (e.g., quality of life) would also be of interest to explore in further studies (e.g., Beierl et al., 2021; Bredemeier, Lieblich, & Foa, 2020). Although the statistical models fit the data well, other variables not measured in this study may have influenced the results and alternative models are also possible. Fourth, like any analysis that investigates changes in constructs over time, this study assumed that the construct that is being measured is the same across treatment sessions. Although there was evidence that scores on these measures are reliable and clinically informative in assessing improvements in symptoms and therapy processes during treatment, the assumption of longitudinal measurement invariance is a limitation and should be tested in larger samples (e.g., Stochl et al., 2020). However, given the short time frame of 10 weekly measurements in our study and the common use of these scales in longitudinal research, we believe the assumption is reasonable.

Future research on processes of change in psychological therapies should also address more directly why therapy does not work for everyone. The focus of this study and the methods used are primarily addressing how therapy works for patients that received at least some core interventions and provided enough data on both symptom and outcome measures to calculate a sufficient number of change scores. While it is likely that the results are also in part relevant for understanding why therapy does not work, investigating factors associated with drop-out or non-recovery would require different methods and different inclusion criteria of participants. Although this study considers nonlinear trajectories of symptoms and the time interval between sessions was consistent across patients, recent advances in methods allow researchers to incorporate differences in time between sessions in the estimation of parameters and should be explored (Driver, Oud, & Voelkle, 2017; Voelkle, Gische, Driver, & Lindenberger, 2018). While this study provides evidence that processes hypothesized by Ehlers and Clark's (2000) model drive clinical improvement, it needs to be further investigated how changes in these processes are related to the therapeutic techniques designed to target them.

Overall, the results of this study provide further evidence that the cognitive and behavioral processes suggested by Ehlers and Clark's (2000) model of PTSD play a key role in driving symptom improvement during CT-PTSD. The results are also consistent with other models (e.g., Foa & Riggs, 1993; Resick & Schnicke, 1992) and highlight potential starting points to improve outcomes. For example, weekly monitoring of changes in theory-derived maintenance factors known to drive symptom change may give therapists and patients a tool to closely track changes and spot early if the interventions are likely to lead to symptom change and adjust interventions accordingly.

## Funding

This study was funded by 10.13039/100010269Wellcome Trust grants 069777 and to 200796 to AE and 10.13039/100014427DC, the Oxford Health NIHR Biomedical Research Centre (NIHR203316) and a Mental Health Research UK studentship awarded to AE and MW. It was supported by the 10.13039/501100015091NIHR South London and Maudsley Biomedical Research Centre. The views expressed are those of the authors and not necessarily those of the NHS, the NIHR or the Department of Health. The funders were not involved in planning or analyzing the study. Open access fees are funded through a block grant to 10.13039/501100000769Oxford University.

## CRediT authorship contribution statement

**Milan Wiedemann:** Conceptualization, Writing – original draft, Methodology, Formal analysis, Visualization. **Magdalena Janecka:** Conceptualization, Formal analysis, Methodology, Writing – review & editing. **Jennifer Wild:** Investigation, Writing – review & editing. **Emma Warnock-Parkes:** Investigation, Writing – review & editing. **Richard Stott:** Investigation, Writing – review & editing. **Nick Grey:** Investigation, Writing – review & editing. **David M. Clark:** Conceptualization, Funding acquisition, Supervision, Writing – review & editing. **Anke Ehlers:** Conceptualization, Funding acquisition, Supervision, Investigation, Writing – review & editing.

## Declaration of competing interest

No potential conflict of interest was reported by the authors.

## Data Availability

Supporting data cannot be made available as patients did not consent to their data being shared. The analysis code is available at https://osf.io/h3v7t.

## Footnotes

1 Pretreatment levels of negative appraisals (PTCI) and disorganized memories (TMQ) were significantly correlated with degree of changes in the corresponding process measures during the first ($\sigma_{j2,ly1\;\left(PTCI\right)}$ = −0.08 [0.03]; $\sigma_{j2,ly1\;\left(TMQ\right)}$ = −0.12 [0.02]) and second ($\sigma_{j3,ly1\;\left(PTCI\right)}$ = −0.04 [0.02]; $\sigma_{j3,ly1\;\left(TMQ\right)}$ = −0.06 [0.02]) part of therapy; pretreatment flashback characteristics (UMQ) only correlated with degree of change in flashback characteristics during the first part of therapy ($\sigma_{j2,ly1\;\left(UMQ\right)}$ = −0.28 [0.11]); and pretreatment unhelpful responses to intrusions (RIQ) and safety behaviors (SBQ) only correlated with changes in the second part of therapy ($\sigma_{j3,ly1\;\left(RIQ\right)}$ = −0.01 [0.00]).
