# Supplementary material for: Changes in cognitive processes and coping strategies precede changes in symptoms during cognitive therapy for posttraumatic stress disorder
Source: Behav Res Ther. 2023 Oct;169:104407. doi: 10.1016/j.brat.2023.104407 (PMC10933802; doi:10.1016/j.brat.2023.104407)
Supplement: Multimedia component 1 [file mmc1.pdf]

Supplemental Online Material

Table A1:

Means and standard deviations for the means of all measures from session 1 to session 10

Session	*n*	PDS	PTCI	TMQ	UMQ	SBQ	RIQ
1	203	1.93 (0.56)	3.95 (1.33)	1.49 (1.15)	5.71 (2.15)	2.01 (0.69)	1.75 (0.55)
2	202	1.80 (0.63)	3.81 (1.29)	1.48 (1.10)	5.42 (2.34)	1.90 (0.72)	1.60 (0.63)
3	207	1.64 (0.63)	3.61 (1.35)	1.41 (1.09)	5.07 (2.33)	1.77 (0.79)	1.47 (0.65)
4	206	1.55 (0.66)	3.30 (1.35)	1.28 (1.02)	4.71 (2.29)	1.77 (0.76)	1.35 (0.64)
5	205	1.34 (0.71)	3.05 (1.38)	1.18 (1.06)	4.09 (2.45)	1.60 (0.80)	1.21 (0.67)
6	204	1.24 (0.74)	2.95 (1.40)	1.02 (0.98)	3.89 (2.49)	1.71 (0.76)	1.13 (0.69)
7	157	1.37 (0.74)	3.14 (1.45)	1.15 (1.03)	4.12 (2.65)	1.66 (0.78)	1.22 (0.69)
8	145	1.22 (0.75)	2.97 (1.39)	0.95 (0.96)	4.03 (2.51)	1.59 (0.79)	1.14 (0.66)
9	132	1.23 (0.79)	2.94 (1.46)	0.96 (0.97)	3.67 (2.69)	1.56 (0.79)	1.10 (0.72)
10	123	1.17 (0.80)	2.87 (1.38)	0.97 (0.99)	3.68 (2.65)	1.48 (0.82)	1.01 (0.72)

Note. Total sample size $n = 217$. PDS = PTSD symptoms; PTCI = Negative appraisals; TMQ = Disorganized memories; UMQ = Flashback characteristics (The scores of this measure were divided by 10 to reduce the variance and facilitate parameter estimation in data analyses); SBQ = Safety behaviors; RIQ = Unhelpful responses to intrusions.

Table A2:

Fit statistics for all univariate latent change score models (best fitting models are highlighted in bold)

Model	χ^2	Parameters	AIC	BIC	CFI	TLI	RMSEA
**PTSD symptoms (PDS)**							
No change	1,546	3	2,900	2,910	.326	.510	.336
Constant change	189	6	1,550	1,570	.941	.955	.102
Piecewise constant change	128	10	1,496	1,530	.967	.973	.079
**Piecewise constant change + AR †**	**119**	**11**	**1,490**	**1,527**	**.970**	**.975**	**.076**
**Negative appraisals (PTCI)**							
No change	1,168	3	4,629	4,639	.507	.643	.290
Constant change	183	6	3,650	3,670	.945	.958	.100
**Piecewise constant change †**	**124**	**10**	**3,599**	**3,632**	**.969**	**.975**	**.077**
Piecewise constant change + AR	121	11	3,598	3,635	.970	.975	.077
**Responses to intrusions (RIQ)**							
No change	1,232	3	2,661	2,671	.382	.552	.296
Constant change	162	6	1,598	1,618	.946	.958	.090
**Piecewise constant change †**	**117**	**10**	**1,560**	**1,594**	**.967**	**.973**	**.072**
Piecewise constant change + AR	116	11	1,561	1,598	.967	.973	.073
**Safety behaviors (SBQ)**							
No change	787	3	2,560	2,570	.621	.725	.235
Constant change	171	6	1,949	1,969	.942	.955	.095
**Piecewise constant change †**	**132**	**10**	**1,919**	**1,952**	**.959**	**.967**	**.082**
Piecewise constant change + AR	132	11	1,920	1,957	.959	.966	.083
**Disorganized memories (TMQ)**							
No change	898	3	3,788	3,798	.574	.691	.252
Constant change	233	6	3,129	3,149	.911	.932	.118
**Piecewise constant change †**	**146**	**10**	**3,049**	**3,083**	**.954**	**.962**	**.088**
Piecewise constant change + AR	143	11	3,048	3,085	.955	.962	.088
**Flashback characteristics (UMQ)**							
No change	902	3	6,679	6,689	.455	.605	.258
Constant change	166	6	5,949	5,968	.931	.947	.094
**Piecewise constant change †**	**131**	**10**	**5,922**	**5,955**	**.951**	**.960**	**.082**
Piecewise constant change + AR	129	11	5,922	5,959	.951	.959	.083

Note. χ^2 = Chi square; AIC = Akaike Information Criterion; BIC = Bayesian Information Criterion; CFI = Comparative Fit Index; TLI = Tucker-Lewis Index; RMSEA = Root Mean Square Error of Approximation. † indicates best fitting model; AR = Autoregressive effect of change scores describing the effect of prior changes on subsequent changes.

Table A3:

Parameter estimates for best fitting univariate latent change score model of PTSD symptoms (PDS)

**Parameter**	*EST* (*SE*)	*p*	EST_{STD}
Initial status mean (γ_{lx1})	1.92 (0.04)	< .001	3.53
Initial status variance (σ^2_{lx1})	0.29 (0.03)	< .001	1
Observed scores variance (σ^2_{ux})	0.06 (0.00)	< .001	0.18
Constant change 1 mean (α_{g2})	-0.10 (0.02)	< .001	-1.13
Constant change 2 mean (α_{g3})	-0.06 (0.02)	.005	-0.96
Constant change 1 variance (σ^2_{g2})	0.01 (0.00)	.004	1
Constant change 2 variance (σ^2_{g3})	0.00 (0.00)	.060	1
Initial status with constant change 1 ($\sigma_{g2,lx1}$)	-0.01 (0.00)	.195	-0.11
Initial status with constant change 2 ($\sigma_{g3,lx1}$)	-0.01 (0.00)	.090	-0.21
Constant change 1 with 2 ($\sigma_{g2,g3}$)	0.00 (0.00)	.396	0.19
Autoregression of change scores (ϕ_x)	0.38 (0.16)	.020	0.27

Note. EST = Unstandardized estimated parameter; SE = Standard error; EST_{STD} = Standardized estimated parameter (completely standardized solution); PDS = Posttraumatic Diagnostic Scale.

Table A4:

Parameter estimates for best fitting univariate latent change score models of process measures

	PTCI			TMQ			UMQ			RIQ			SBQ		
**Parameter**	*EST* (*SE*)	*p*	EST_{STD}	*EST* (*SE*)	*p*	EST_{STD}	*EST* (*SE*)	*p*	EST_{STD}	*EST* (*SE*)	*p*	EST_{STD}	*EST* (*SE*)	*p*	EST_{STD}
Initial status mean (γ_{ly1})	4.07 (0.09)	< .001	3.21	1.58 (0.08)	< .001	1.41	5.93 (0.16)	< .001	2.89	1.78 (0.04)	< .001	3.34	2.02 (0.05)	< .001	3.14
Initial status variance (σ^2_{ly1})	1.60 (0.15)	< .001	1	1.25 (0.10)	< .001	1	4.22 (0.47)	< .001	1	0.28 (0.03)	< .001	1	0.41 (0.05)	< .001	1
Observed scores variance (σ^2_{uy})	0.23 (0.02)	< .001	0.12	0.16 (0.01)	< .001	0.12	1.21 (0.12)	< .001	1.22	0.07 (0.01)	< .001	0.20	0.09 (0.01)	< .001	0.18
Constant change 1 *mean* (α_{j2})	-0.22 (0.02)	< .001	-0.90	-0.09 (0.02)	< .001	-0.43	-0.39 (0.04)	< .001	-0.86	-0.13 (0.01)	< .001	-1.10	-0.08 (0.01)	< .001	-0.68
Constant change 2 *mean* (α_{j3})	-0.14 (0.01)	< .001	-0.98	-0.11 (0.01)	< .001	-0.84	-0.32 (0.03)	< .001	-1.17	-0.08 (0.01)	< .001	-1.09	-0.08 (0.01)	< .001	-0.76
Constant change 1 *variance* (σ^2_{j2})	0.06 (0.01)	< .001	1	0.04 (0.01)	< .001	1	0.20 (0.04)	< .001	1	0.01 (0.00)	< .001	1	0.01 (0.00)	< .001	1
Constant change 2 *variance* (σ^2_{j3})	0.02 (0.01)	< .001	1	0.02 (0.00)	< .001	1	0.07 (0.02)	< .001	1	0.01 (0.00)	< .001	1	0.01 (0.00)	< .001	1
Initial status with constant change 1 ($\sigma_{j2,ly1}$)	-0.08 (0.03)	.001	-0.26	-0.12 (0.02)	< .001	-0.51	-0.28 (0.11)	.011	-0.30	-0.01 (0.01)	.119	-0.15	-0.01 (0.01)	.454	-0.09
Initial status with constant change 2 ($\sigma_{j3,ly1}$)	-0.04 (0.02)	.042	-0.24	-0.06 (0.02)	< .001	-0.41	-0.01 (0.08)	.862	-0.02	-0.01 (0.00)	.038	-0.24	-0.01 (0.01)	.038	-0.21
Constant change 1 *with* 2 ($\sigma_{j2,j3}$)	0.01 (0.01)	.008	0.38	0.00 (0.00)	.386	0.12	0.03 (0.02)	.134	0.24	0.00 (0.00)	.015	0.38	0.00 (0.00)	.777	0.06

Note. EST = Unstandardized estimated parameter; SE = Standard error; EST_{STD} = Standardized estimated parameter (completely standardized solution); PTCI = Negative appraisals; TMQ = Disorganized memories; UMQ = Flashback characteristics; RIQ = Unhelpful responses to intrusions; SBQ = Safety behaviors

Table A5:

Fit statistics for all bivariate latent change score models (best fitting models are highlighted in bold)

**Model**	χ^2	**Parameters**	**AIC**	**BIC**	**CFI**	**TLI**	**RMSEA**
**Negative appraisals - PTSD symptoms**							
No Coupling	644	23	4,695	4,772	.915	.922	.100
$\Delta Negative\ appraisals_{(t-1)} \rightarrow \Delta PTSD\ symptoms_{(t)}$	420	24	4,473	4,554	.958	.962	.070
$\Delta PTSD\ symptoms_{(t-1)} \rightarrow \Delta Negative\ appraisals_{(t)}$	435	24	4,487	4,568	.956	.959	.072
$\boldsymbol{Bidirectional\ coupling^{\dagger}}$	**410**	**25**	**4,465**	**4,549**	**.960**	**.963**	**.069**
**Unhelpful responses to intrusions - PTSD symptoms**							
No Coupling	620	23	2,657	2,735	.914	.922	.096
$\Delta Responses\ to\ intrusions_{(t-1)} \rightarrow \Delta PTSD\ symptoms_{(t)}$	374	24	2,413	2,494	.965	.968	.062
$\Delta PTSD\ symptoms_{(t-1)} \rightarrow \Delta Responses\ to\ intrusions_{(t)}$	429	24	2,468	2,549	.954	.957	.071
$\boldsymbol{Bidirectional\ coupling^{\dagger}}$	**373**	**25**	**2,414**	**2,498**	**.965**	**.968**	**.062**
**Safety behaviors - PTSD symptoms**							
No Coupling	520	23	3,193	3,270	.931	.936	.085
$\boldsymbol{\Delta Safety\ behaviors_{(t-1)} \rightarrow \Delta PTSD\ symptoms^{\dagger}_{(t)}}$	**389**	**24**	**3,064**	**3,144**	**.959**	**.963**	**.065**
$\Delta PTSD\ symptoms_{(t-1)} \rightarrow \Delta Safety\ behaviors_{(t)}$	418	24	3,093	3,174	.953	.957	.070
$Bidirectional\ coupling$	389	25	3,066	3,150	.959	.962	.065
**Disorganized memories - PTSD symptoms**							
No Coupling	477	23	4,319	4,396	.939	.944	.078
$\Delta Disorganized\ memories_{(t_{1,2}-1)} \rightarrow \Delta PTSD\ symptoms_{(t_{1,2})}$	407	25	4,252	4,336	.954	.958	.068
$\Delta PTSD\ symptoms_{(t_{1,2}-1)} \rightarrow \Delta Disorganized\ memories_{(t_{1,2})}$	368	25	4,214	4,298	.963	.966	.061
$\boldsymbol{Piecewise\ bidirectional\ coupling^{\dagger}}$	**364**	**27**	**4,214**	**4,304**	**.964**	**.966**	**.061**
**Flashback characteristics - PTSD symptoms**							
No Coupling	595	24	6,897	6,976	.911	.918	.096
$\Delta Flashback\ characteristics_{(t_{1,2}-1)} \rightarrow \Delta PTSD\ symptoms_{(t_{1,2})}$	384	26	6,689	6,776	.959	.962	.066
$\Delta PTSD\ symptoms_{(t_{1,2}-1)} \rightarrow \Delta Flashback\ characteristics_{(t_{1,2})}$	365	26	6,671	6,757	.963	.966	.062
$\boldsymbol{Piecewise\ bidirectional\ coupling^{\dagger}}$	**356**	**27**	**6,664**	**6,754**	**.965**	**.967**	**.061**

Note. χ^2 = Chi Square; AIC = Akaike Information Criterion; BIC = Bayesian Information Criterion; CFI = Comparative Fit Index; TLI = Tucker-Lewis Index; RMSEA = Root Mean Square Error of Approximation. † indicates best fitting model.

Table A6:

Pearson correlations of PTSD symptoms and cognitive process measures at session 1

	PDS	PTCI	RIQ	SBQ	UMQ	TMQ
PDS	–					
PTCI	0.69**	–				
RIQ	0.63**	0.65**	–			
SBQ	0.47**	0.48**	0.54**	–		
UMQ	0.66**	0.56**	0.51**	0.46**	–	
TMQ	0.36**	0.42**	0.32**	0.14	0.37**	–

Note. Sample size $n = 217$. ** statistically significant at $p < .001$. PDS = PTSD symptoms; PTCI = Negative appraisals; TMQ = Disorganized memories; UMQ = Flashback characteristics; SBQ = Safety behaviors; RIQ = Unhelpful responses to intrusions.
